# Liquid chromatography–tandem mass spectrometry method for mycophenolic acid and its glucuronide determination in saliva samples from children with nephrotic syndrome

**DOI:** 10.1007/s43440-024-00574-9

**Published:** 2024-03-15

**Authors:** Joanna Sobiak, Matylda Resztak, Weronika Sikora, Jacek Zachwieja, Danuta Ostalska-Nowicka

**Affiliations:** 1 Department of Physical Pharmacy and Pharmacokinetics, Poznan University of Medical Sciences, 3 Rokietnicka Street, 60-806 Poznan, Poland; 2 Department of Pediatric Nephrology and Hypertension, Poznan University of Medical Sciences, Poznan, Poland

**Keywords:** Mycophenolate mofetil, Therapeutic drug monitoring, Saliva, Nephrotic syndrome, Pediatric patients

## Abstract

**Background:**

Saliva sampling is one of the methods of therapeutic drug monitoring for mycophenolic acid (MPA) and its metabolite, mycophenolic acid glucuronide (MPAG). The study describes the liquid chromatography tandem mass spectrometry (LC–MS/MS) method developed for saliva MPA and MPAG determination in children with nephrotic syndrome.

**Methods:**

The mobile phase consisted of methanol and water at gradient flow, both with 0.1% formic acid. Firstly, 100 µL of saliva was evaporated at 45 °C for 2 h to dryness, secondly, it was reconstituted in the mobile phase, and finally 10 µL was injected into the LC–MS/MS system. Saliva from ten children with nephrotic syndrome treated with mycophenolate mofetil was collected with Salivette^®^.

**Results:**

For MPA and MPAG, within the 2–500 ng/mL range, the method was selective, specific, accurate and precise within-run and between-run. No carry-over and matrix effects were observed. Stability tests showed that MPA and MPAG were stable in saliva samples if stored for 2 h at room temperature, 18 h at 4 °C, and at least 5 months at − 80 °C as well as after three freeze–thaw cycles, in a dry extract for 16 h at 4 °C, and for 8 h at 15 °C in the autosampler. The analytes were not adsorbed onto Salivette^®^ cotton swabs. For concentrations above 500 ng/mL, the samples may be diluted twofold. In children, saliva MPA and MPAG were within the ranges of 4.6–531.8 ng/mL and 10.7–183.7 ng/mL, respectively.

**Conclusions:**

The evaluated LC–MS/MS method has met the validation requirements for saliva MPA and MPAG determination in children with nephrotic syndrome. Further studies are needed to explore plasma–saliva correlations and assess their potential contribution to MPA monitoring.

**Graphical abstract:**

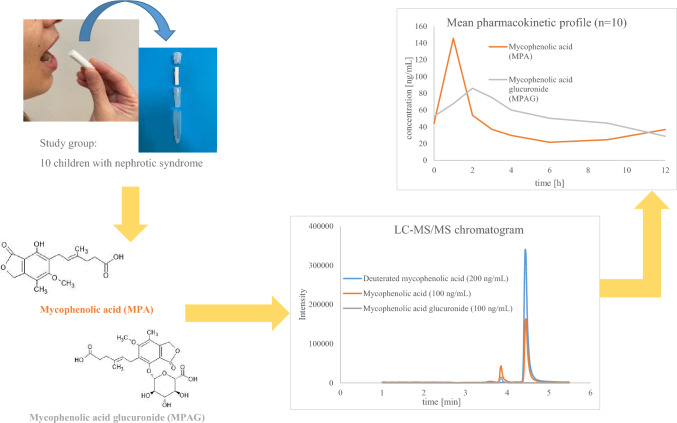

**Supplementary Information:**

The online version contains supplementary material available at 10.1007/s43440-024-00574-9.

## Introduction

Mycophenolic acid (MPA) is the active form of mycophenolate mofetil (MMF). Initially, MMF was registered as a therapeutic agent in acute rejection prophylaxis after renal transplantation, but it is now prescribed also in other diseases, such as nephrotic syndrome, lupus nephritis, and atopic dermatitis [1–3]. There are several indications for therapeutic drug monitoring (TDM) of MPA, such as its variable pharmacokinetics with a second maximum concentration (C_max2_) occurrence, a high level of albumin binding (97–99%), and its dependence on renal function [1, 2]. The occurrence of C_max2_ is related to the enterohepatic recirculation of the inactive MPA metabolite, mycophenolic acid glucuronide (MPAG), which is converted back to MPA [2]. It was previously postulated that in children with nephrotic syndrome, the target values of the area under the curve from 0 to 12 h (AUC_0–12_) should be higher than those after renal transplantation to ensure effective therapy [4–6].

Although TDM is not routine in the case of MPA, some studies have demonstrated its utility, particularly in children [7–9]. An advantageous matrix for studying the pediatric population is saliva, which is easy to obtain with significantly lower stress levels compared to blood, the most common biological matrix for TDM [10]. Saliva collection is a non-invasive and straightforward process that does not involve time-consuming procedures [11, 12]. Furthermore, saliva contains various molecules derived from blood, including drugs and their metabolites, enabling the monitoring of drug concentrations in the patient's body [13]. Moreover, saliva concentrations reflect the unbound drug levels, which are pharmacologically active [14, 15]. The use of saliva as an analytical matrix offers numerous advantages, but it also presents challenges, particularly in developing analytical methods that meet validation requirements for accurate and precise determination of low drug concentrations in saliva.

MPA is classified as a class III compound in the Salivary Excretion Classification System (SECS III), which makes it suitable for saliva determination [14, 16]. Since children with nephrotic syndrome require higher target MPA AUC_0–12_ values than renal transplant recipients, we searched for the effective method of facilitating MPA TDM in this group of patients. One option include determining MPA and MPAG in saliva using the liquid chromatography tandem mass spectrometry (LC–MS/MS) method. While several LC–MS/MS methods for saliva MPA determination are described in the literature [14, 15, 17–20], to our knowledge, only two include MPAG analysis [17, 20]. Mendonza et al. [18] mentioned MPAG in their study, but focused solely on saliva MPA levels. MPAG determination is crucial for monitoring therapy effectiveness and MPA pharmacokinetics as Hui-Yuen et al. [21] suggested that MPAG could serve as a marker for MMF absorption and metabolism. Some studies have shown that saliva MPA and MPAG concentrations might be alternatives to plasma measurements due to observed correlations. However, other studies suggest that saliva concentrations do not necessarily reflect plasma levels, particularly in renal transplant recipients, whose kidney function might influence MPA concentrations [15, 22]. This disparity underscores the importance of assessing the range of MPA concentrations and the existence of saliva-plasma correlations in children with nephrotic syndrome.

In our previous study [23], we successfully applied high-performance liquid chromatography with fluorescence detection (HPLC-FLD) method for MPA determination in saliva, but were unable to detect MPAG. Consequently, we explored the possibility of determining both MPA and MPAG in saliva using the liquid chromatography tandem mass spectrometry (LC–MS/MS) method.

## Materials and methods

### Analytical standards and reagents

All solvents and reagents were of LC–MS grade. MPA (powder, Product No. M5255) and deuterated MPA (MPA-d3, internal standard [IS], liquid, product No. M-137) and deuterated MPAG (MPAG-d3, liquid, product No. M-204) were purchased from Sigma-Aldrich (Germany). MPAG was purchased from LGC Standards (solid, product No. TRC-M831520, Toronto Research Chemicals). Hypergrade for LC–MS methanol (Product No. 1.06035), acetonitrile (Product No. 1.00029.1000), and water (Product No. 1.15333) were obtained from Merck (Germany).

### Standard solutions

Stock solutions of 1000 μg/mL of MPA and 1000 μg/mL of MPAG were prepared by dissolving the specified quantities of the compounds in acetonitrile. The IS was purchased as 100 μg/mL solution in acetonitrile. Calibration standards, prepared from stock solutions by dilution, were within the concentration range of 4–1000 ng/mL for both MPA and MPAG working solutions. The concentration of the IS working solution was set at 400 ng/mL. All stock and working solutions were stored at − 80 °C.

### Chromatographic conditions and apparatus

The analysis was performed in the Shimadzu Ultra-Performance Liquid Chromatography (UPLC) set (Shimadzu Co., Kyoto, Japan), equipped with a solvent degasser (DGU-20A5), a binary pump (LC-20AD), a thermostated autosampler (SIL-30AC), and a column compartment (CTO-20AC). UPLC was conjoined with a triple quadrupole detector LCMS-8030. Data processing was carried out using the LabSolutions Series Workstation system (Shimadzu, Kyoto, Japan).

The mobile phase was composed of methanol with 0.1% formic acid and water with 0.1% formic acid, in a gradient flow (the content of methanol with 0.1% formic acid: 0–1.5 min 20% 1.5–3.0 min 20–70%; 3.0–5.5 min 70%; 5.5–6.0 min 70–20%; 6.0–6.5 min 20%). The flow rate was set at 0.5 mL/min, and the temperature for chromatographic separation was maintained at 40 °C. Zorbax Eclipse Plus C18 (2.1 mm × 100 mm, 3.5 μm, Agilent, USA), with a guard column Eclipse Plus C18 Grd (2.1 × 12.5 mm, 5 μm, Agilent, USA), were used for MPA and MPAG determination. Positive electrospray ionization mode (ESI+) was used for MPA and MPA-d3, while negative electrospray ionization mode (ESI−) was used for MPAG. The injection volume was 10 µL. The desolvation line and the heat block temperature were maintained at 245 °C and 400 °C, respectively. Nitrogen was used as the drying gas and nebulizing gas, with flow rates of 12 and 2 L/min, respectively, and argon was used as the collision gas. The electrospray needle voltage was at 4.5 kV. Multiple reaction monitoring mode was employed to identify the most sensitive mass transition, which were observed at *m*/*z* 321.2 > 303.05 and 321.2 > 206.9 for MPA, 495.2 > 319.25 and 495.2 > 191.05 for MPAG, and 324.1 > 306.05 and 324.1 > 209.9 for MPA-d3.

### Samples preparation

A volume of 100 µL of blank saliva was mixed with 50 µL of working solutions and 50 µL of IS working solution. MPA concentrations in the matrix were as follows: 2, 5, 10, 20, 50, 100, 200, and 500 ng/mL, and an IS concentration was 200 ng/mL. After mixing, the samples were evaporated to dryness at 45 °C for 2 h in a centrifugal vacuum concentrator (Eppendorf, Germany). The dry extract was reconstituted in 100 µL of mobile phase (methanol and water mixed in a 20:80 ratio, both containing 0.1% formic acid), transferred to Eppendorf tubes and subsequently centrifuged twice for 10 min at 16,000×*g*.

### Method validation

The developed method was validated based on the guidelines on bioanalytical method validation of the European Medicines Agency [24] and covered aspects such as selectivity, specificity, calibration curves, within-run and between-run precision and accuracy, carry-over, matrix effect, dilution integrity, and sample stability under various conditions. We also checked the recovery of the analyzed substances from the cotton swabs.

### Selectivity and specificity

Selectivity was evaluated using seven different specimens of blank saliva, collected from healthy adult volunteers. Interfering components should not exceed 20% of the analyte response at the lower limit of quantification (LLOQ) and 5% of the IS response. Specificity was assessed to determine whether the method can detect and differentiate MPA, MPAG, and IS from a few drugs that may be co-administered with MMF, such as paracetamol, voriconazole, itraconazole, ketoconazole, and amlodipine.

### The lower limit of quantification (LLOQ)

The LLOQ was defined as the lowest concentration of MPA and MPAG that can be quantitatively determined using a method with predefined precision and accuracy. The accuracy of the back-calculated concentration at the LLOQ should be within ± 20% of the nominal concentration, and the precision of the LLOQ (expressed as coefficient of variation, %CV) should not exceed 20% [24].

### Calibration curve

The calibration curve for MPA was constructed by plotting the ratio of the MPA peak area to the IS peak area against the nominal concentrations of MPA (P_MPA_/P_IS_ = f(C_MPA_)). The calibration curve for MPAG was constructed by plotting the MPAG peak area against the MPAG nominal concentrations (P_MPAG_ = f(C_MPAG_)). Each calibration curve was analyzed using linear regression and an unweighted power function was implemented. The correlation coefficient r was calculated and the Student’s t-test was applied for the evaluation of the linearity. The example of MPA and MPAG calibration curves with relevant equations are presented in Supplementary Figure S1 (A and B) and Supplementary Table S1.

### Accuracy and precision

Within-run and between-run accuracy and precision were calculated for the LLOQ, low (5 ng/mL), medium (200 ng/mL), and high (500 ng/mL) quality controls (QCs) samples (5 replicates of each). The accuracy was calculated as C_measured_/C_nominal_·100%. Precision was expressed as %CV.

### Carry-over

Carry-over was assessed by injecting five replicates of blank saliva samples following the calibration standard of MPA and MPAG at the upper limit of quantification (ULOQ, high QC). The absence of the carry-over effect was confirmed when the response of the MS-detector was below 20% for the analyte response at the LLOQ and below 5% for IS at the respective retention times.

### Matrix effect

The matrix effect was evaluated by analyzing three replicates of low and high QCs, each prepared using saliva from seven volunteers. The absence of matrix effect was confirmed if the accuracy was within ± 15% of the nominal concentration and the %CV did not exceed 15%.

### Dilution integrity

Dilution integrity was assessed using five replicates of saliva samples prepared with MPA and MPAG at concentrations of 800 ng/mL, which were diluted twofold with blank saliva to achieve a concentration of 400 ng/mL for both analytes. The mean accuracy of these diluted samples should be within ± 15% of the nominal concentration, and the %CV should not exceed 15%.

### Stability

The stability of MPA and MPAG in saliva was assessed when stored for 2 h at room temperature (20 °C), for 18 h at 4 °C, and through three freeze–thaw cycles. Long-term stability was evaluated for samples stored at − 80 °C for 5 months. The stability of the processed samples was assessed under various storage conditions, including dry extract storage for 16 h at 4 °C, and the stability of analytical samples in the autosampler for 8 h at 15 °C. In each stability test, three replicates of QCs at low and high concentrations were analyzed against a calibration curve from freshly spiked calibration standards. The mean concentration at both QC levels should be within ± 15% of the nominal concentration.

### Cotton swabs adsorption

The adsorption of MPA and MPAG onto the plain cotton swab from Salivette^®^ was determined using a recovery test. Initially, unstimulated saliva was obtained from three healthy volunteers. Subsequently, MPA and MPAG QCs at low and high concentrations were prepared in centrifuged unstimulated saliva and spiked on the Salivette^®^ swabs. The Salivette^®^ devices were incubated for 5 min at 37 °C to simulate the time of saliva collection, and prepared for the LC–MS/MS analysis according to the above-described sample handling scheme. Three replicates of each concentration were analyzed. The recovery was assessed as a percentage of the nominal MPA and MPAG concentrations.

### In vivo application

The developed analytical method was applied to determine MPA and MPAG concentrations in saliva samples collected from ten pediatric patients (aged 9.1 ± 3.8 years; six boys and four girls) with nephrotic syndrome treated with MMF. The mean height and body weight were 133.8 ± 19.8 cm and 32.6 ± 15.4 kg, respectively. The children were treated in the Department of Pediatric Nephrology and Hypertension at Poznan University of Medical Sciences (Poznań, Poland) with a consistent MMF dose for at least 1 month before saliva collection. MMF was administered twice a day, with median morning and evening doses of 500 mg. The range of MMF single doses was within 300–1000 mg. The study was approved by the Ethical Committee at Poznan University of Medical Sciences (Decisions numbers 773/21 and 808/22).

Saliva samples were collected using Salivette^®^ devices with a cotton swab at the following time points: before the administration of the next MMF dose (C_trough_), and subsequently 1 h (C_1_), 2 h (C_2_), 3 h (C_3_), 4 h (C_4_), 6 h (C_6_), 9 h (C_9_), 12 h (C_12_) afterwards. According to the manufacturer’s instruction, the swab was kept and chewed in the mouth for 1 min. After immediate centrifuging (2 min, 1000×*g*), the collected saliva was stored at 4 °C for a maximum of 6 h and then transported and stored at − 80 °C until analysis.

For MPA and MPAG, the following pharmacokinetic parameters were calculated: maximum concentration (C_max_), time to reach maximum concentration (T_max_), C_max2_, time to reach C_max2_ (t_max2_), and AUC_0–12_ using the trapezoid method. C_max2_ was calculated for MPA as the 20% increase in its concentration when compared to the concentration determined in the preceding sample. The results were presented as mean ± standard deviation (SD), except for T_max_, for which the median value and range were provided.

The results of the LC–MS/MS method were compared with those obtained using the HPLC-FLD method, which was previously developed [23]. Since MPAG concentrations were not determined in the previous study, and only two children (one boy and one girl, both aged 12 years) were included in the MPA analysis, we limited our comparison to these specific cases, encompassing 16 MPA concentrations. These results are presented in Supplementary Figure S2 (A and B).

### Statistical analysis

All statistical tests were performed using Statistica software version 13 (StatSoft, Cracow, Poland). Normality was determined by the Shapiro–Wilk test. The correlation of the data was tested using Pearson correlation analysis as the analyzed data were normally distributed. To compare results from the LC–MS/MS method with those obtained using the HPLC-FLD method [23], we assessed Passing-Bablok regression and Bland–Altman plot. In all analyses, a p-value of 0.05 was considered significant.

## Results

### Validation results

**Fig. 1:**
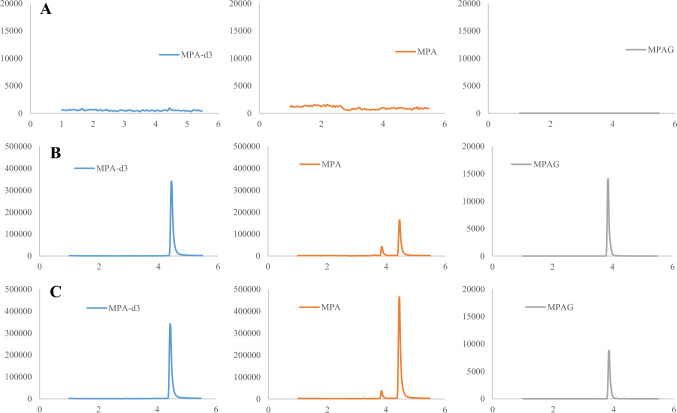
The LC–MS/MS chromatograms of **A** blank saliva, **B** saliva spiked with mycophenolic acid (MPA) and mycophenolic acid glucuronide (MPAG) (both at a concentration of 100 ng/mL in matrix) and internal standard (IS, 200 ng/mL in matrix), **C** saliva collected 1 h after drug administration from a child treated with mycophenolate mofetil (MMF). Responses from deuterated MPA (MPA-d3; internal standard, IS), MPA, and MPAG are represented by blue, orange and grey lines, respectively

The developed LC–MS/MS method demonstrated high selectivity and specificity, as no interfering peaks from endogenous substances or the analyzed drugs were observed. Exemplary chromatograms of a blank sample, a sample spiked with MPA, MPAG, and IS, and a saliva sample collected from a child 1 h after MMF administration are presented in Fig. 1. The average retention times for MPA, MPAG, and IS were 4.5, 3.9, and 4.4 min, respectively.

The method was linear within the 2–500 ng/mL range for both MPA and MPAG, with LLOQ of 2 ng/mL. The accuracy and precision fulfilled the acceptance criteria for within-run and between-run analyses (Table 1). For MPA and MPAG calibration curves, the intercept values were not significant (Student’s test; α = 0.05).

**Table 1:** Within-run and between-run accuracy, calculated as C_measured_/C_nominal_·100%, and precision, expressed as the coefficient of variation (%CV), calculated for MPA and MPAG at four quality control levels

QC concentration (ng/mL)	Accuracy (%)	Precision (%CV)	Accuracy (%)	Precision (%CV)
	Within-run		Between-run	
	n = 5		n = 5	
MPA				
2 (LLOQ)	99.1	5.8	92.5	14.6
5 (low QC)	93.9	5.8	97.6	11.9
200 (medium QC)	106.2	1.6	100.1	8.8
500 (ULQC)	98.1	1.6	100.2	1.5
MPAG				
2 (LLOQ)	114.7	3.4	100.9	12.0
5 (low QC)	95.1	10.6	96.9	11.7
200 (medium QC)	108.2	2.1	100.4	4.3
500 (ULQC)	106.4	3.4	99.9	0.5

*CV* coefficient of variation, *LLOQ* lower limit of quantitation, *MPA* mycophenolic acid, *MPAG* mycophenolic acid glucuronide, *QC* quality control, *ULQC* upper limit quality control

Matrix effects, often caused by alterations in the ionization efficiency of target analytes in the presence of co-eluting compounds in the same matrix, can result in either a loss in response (ion suppression) or an increase in response (ion enhancement) [25, 26]. In our study, we did not observe either the carry-over effect or the matrix effect. The accuracy for the matrix effect for MPA and MPAG was within ± 15% of the nominal concentration and the precision did not exceed 15% for all seven matrices and both concentrations studied (Table 2). These results indicate that matrix components are successfully removed and do not affect the method’s performance. The results of the dilution integrity test indicated that saliva samples with MPA and MPAG above ULOQ may be diluted twice. The mean accuracy was 9.1% and 9.6% for MPA and MPAG, respectively, while the precision was 2.7% and 2.9% for MPA and MPAG, respectively.

**Table 2:** Matrix effect results, accuracy (calculated as C_measured_/C_nominal_·100%) and precision (expressed as the coefficient of variation, %CV), calculated using blank saliva from seven healthy volunteers

Matrix no.	MPA		MPAG	
	Accuracy (%)	Precision (%CV)	Accuracy (%)	Precision (%CV)
5 ng/mL				
1	90.7	6.1	95.6	10.1
2	90.6	2.7	101.3	1.4
3	97.7	1.5	111.7	3.3
4	105.2	2.4	106.4	3.1
5	95.5	9.0	112.4	3.3
6	101.9	2.4	106.6	1.0
7	95.8	1.9	108.8	6.3
500 ng/mL				
1	106.3	5.1	107.4	0.9
2	100.2	0.4	96.3	1.4
3	111.1	2.9	101.6	2.7
4	110.5	2.2	102.5	5.5
5	112.1	1.4	109.2	1.7
6	104.7	2.7	109.7	2.5
7	108.8	5.2	113.7	1.5

*%CV* coefficient of variation, *MPA* mycophenolic acid, *MPAG* mycophenolic acid glucuronide

MPA and MPAG in saliva samples were stable if stored at room temperature for 2 h, at 4 °C for 18 h, and after 5 months of storage at − 80 °C. The results confirmed that saliva samples with MPA and MPAG content may be frozen and thawed three times, and the dry residue may be stored at 4 °C for 16 h. MPA and MPAG analytical samples could be analyzed within 8 h after placement in vials in an autosampler thermostated at 15 °C. The stability test results for MPA and MPAG are shown in Table 3.

**Table 3:** Stability (expressed in % as a mean relative error) of mycophenolic acid (MPA) and mycophenolic acid glucuronide (MPAG), at low (5 ng/mL) and high quality control (500 ng/mL) levels, in saliva, in processed samples and in the analytical samples stored in various conditions. All results were calculated from three replicates of each sample

Analyte	Nominal concentration (ng/mL)	Saliva samples				Processed samples	Analytical samples
		2 h, room temperature (20 °C)	18 h, 4 °C	4 weeks, − 80 °C	Freeze–thaw (3 cycles)	Dry residue 16 h, 4 °C	8 h in Autosampler, 15 °C
MPA	5	99.8	90.0	101.3	100.3	98.5	95.9
	500	99.0	99.8	94.3	95.6	109.7	107.5
MPAG	5	106.9	99.5	105.2	100.9	92.5	99.5
	500	100.3	97.3	94.9	95.4	93.2	100.8

*MPA* mycophenolic acid, *MPAG* mycophenolic acid glucuronide

MPA and MPAG at low and high QC concentrations were not adsorbed considerably onto the cotton swabs of Salivette^®^. The relative errors for low QC samples spiked on the cotton swabs were 90.9% and 100.7% for MPA and MPAG, respectively, and for ULOQ 101.6% and 100.1% for MPA and MPAG, respectively. The %CV was 5.0% and 5.9% for MPA low QC and ULOQ, respectively, and 9.5% and 9.3% for MPAG low QC and ULOQ, respectively.

### Application

**Fig. 2:**
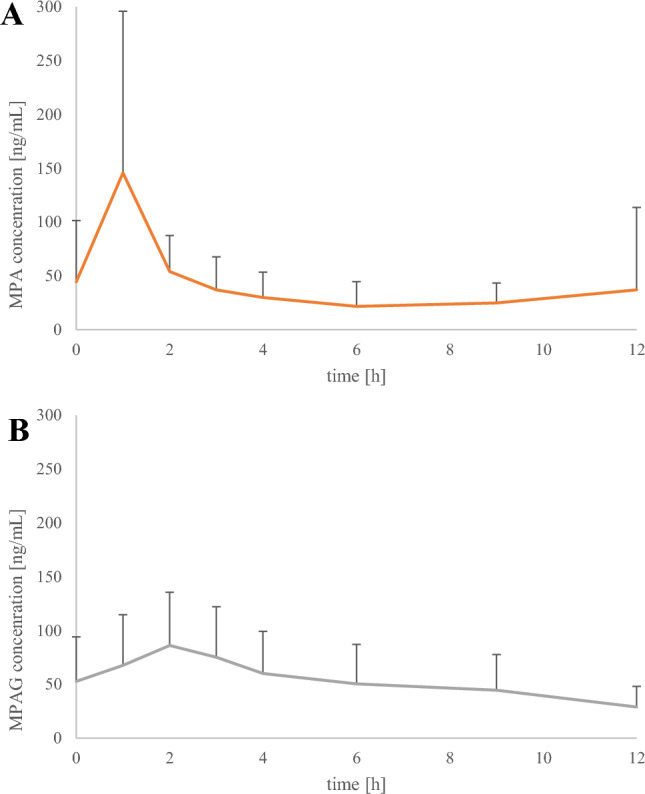
Mean pharmacokinetic profiles with standard deviation (+ SD) of saliva mycophenolic acid (MPA; **A**) and saliva mycophenolic acid glucuronide (MPAG; B) determined in ten children treated with mycophenolate mofetil (MMF) within 12 h after administration of a single dose

**Fig. 3:**
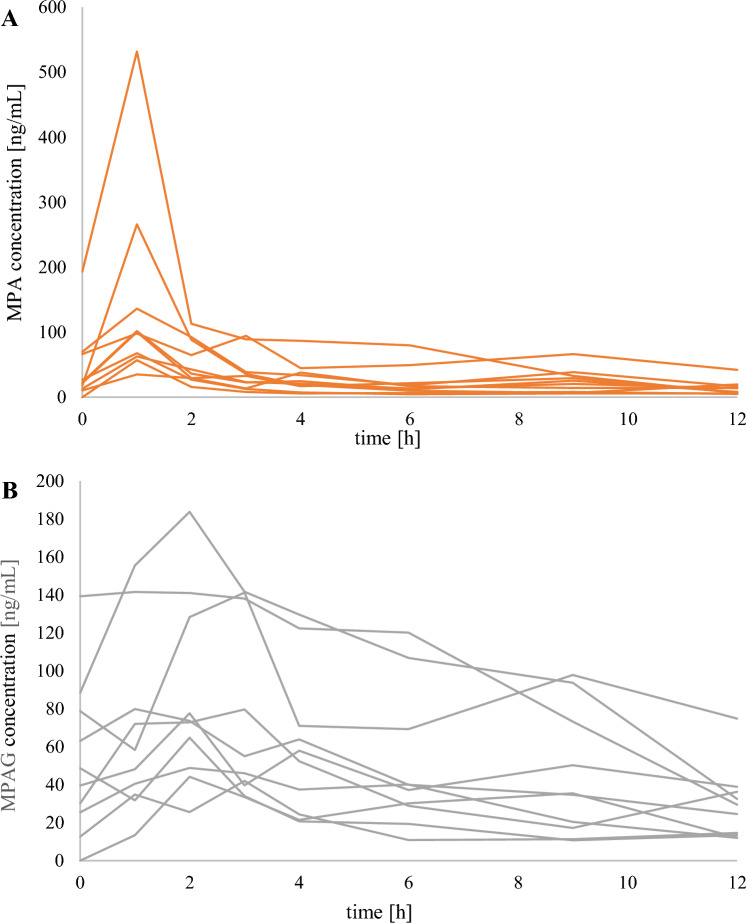
Individual saliva pharmacokinetic profiles of mycophenolic acid (MPA; **A**) and mycophenolic acid glucuronide (MPAG; **B**) determined in ten children treated with mycophenolate mofetil (MMF) within 12 h after administration of a single dose

The method was successfully applied to determine MPA and MPAG saliva concentrations in ten children with nephrotic syndrome treated with MMF. The mean pharmacokinetic profiles of saliva MPA and MPAG, with SD, are presented in Fig. 2, whereas ten individual pharmacokinetic profiles are presented in Fig. 3. MPA concentrations ranged from 4.6 to 531.8 ng/mL, with only one sample exceeding the ULOQ (500 ng/mL). The second-highest saliva MPA concentration was 265.9 ng/mL. MPAG saliva concentrations varied from 10.7 to 183.7 ng/mL. In one child, possible non-adherence was observed, as MPA and MPAG were undetectable in the pre-dose sample, suggesting that the child might not have received the evening dose of MMF, or more previous doses, or that the drug was not administered at all. The mean saliva MPA C_max_ was 145.6 ± 150.2 ng/mL, and it was observed 1 h after MMF administration in all children. In one child, MPA C_max2_ was not indicated, whereas in nine children MPA C_max2_ was observed within 4–12 h with a median t_max2_ of 9.0 h and amounted to 27.9 ± 18.3 ng/mL. Mean MPA and MPAG pharmacokinetic parameters (e.g. C_max_, t_max_, AUC_0–12_) are presented in Table 4. The median MPA and MPAG AUC_0–12_ amounted to 339 ng h/mL (range 138–1268 ng h/mL) and 535 ng h/mL (range 228–1316 ng h/mL).

**Table 4:** Mean saliva mycophenolic acid (MPA) and mycophenolic acid glucuronide (MPAG) concentrations and pharmacokinetic parameters in ten children with nephrotic syndrome treated with mycophenolate mofetil (MMF)

	C_trough_	C_1_	C_2_	C_3_	C_4_	C_6_	C_9_	C_12_
Saliva MPA concentration [ng/mL]								
Mean	44.2	145.6	53.8	37.0	29.8	21.6	24.7	36.9
SD	57.1	150.2	33.5	30.6	23.6	22.9	18.5	76.7
CV%	129	103	62	83	79	106	75	208
Saliva MPAG concentration [ng/mL]								
Mean	52.6	67.6	86.0	75.1	60.1	50.3	44.5	28.9
SD	41.3	47.0	49.4	46.9	39.1	36.8	33.1	19.2
CV%	79	69	57	62	65	73	74	66

	Saliva MPA					Saliva MPAG		
	AUC_0–12_ [ng h/mL]	C_max_ [ng/mL]	t_max_ [h]	C_max2_^c^ [ng/mL]	t_max2_ [h]	AUC_0–12_ [ng h/mL]	C_max_ [ng/mL]	t_max_ [h]
Mean^a^	457	145.6	1	27.9	9	637	90.3	2
SD^b^	339	150.2	1–1	18.3	4–12	385	48.5	1–3
CV%	74	103	–	66	–	60	54	-

*AUC*_*0–12*_ area under the concentration–time from 0 to 12 h curve, *C*_*max*_ maximum concentration,* C*_*max2*_ secondary maximum concentration, *C*_*trough*_*, C*_*1*_*, C*_*2*_*, C*_*3*_*, C*_*4*_*, C*_*6*_*, C*_*9*_*, C*_*12*_ concentrations determined pre-dose, 1, 2, 3, 4, 6, 9, and 12 h after dosing, respectively, *MMF* mycophenolate mofetil, *MPA* mycophenolic acid, *MPAG* mycophenolic acid glucuronide, *SD* standard deviation, *CV%* coefficient of variation, *t*_*max*_ time of maximum concentration, *t*_*max2*_ time of the second maximum concentration

^a^Median value for t_max_

^b^Range for t_max_ and t_max2_

^c^n = 9

The MPA saliva concentrations determined with LC–MS/MS and HPLC-FLD methods were strongly correlated (r = 0.9690, p < 0.0001). The Bland–Altman plot (Supplementary Fig. S2A) showed that the results of HPLC-FLD analysis are lower than those from LC–MS/MS, as the mean difference was − 3.661. The line of equality was within the confidence interval of the mean difference, suggesting an insignificant bias. Out of 16 MPA concentrations, one concentration (6%) lay outside the ± confidential interval, indicating erratic variability as it exceeds 5% [27]. In the Passing-Bablok regression analysis (Supplementary Fig. S2B), the 95% confidence interval (CI) for slope included 1 (slope: 0.9345, 95% CI 0.8047–1.0818), and the 95% CI for intercept included 0 (intercept: 0.1539, 95% CI − 5.7301 to 4.997), suggesting no proportional or constant differences between the methods [28].

## Discussion

In this study, we validated the LC–MS/MS method for both MPA and MPAG determination in saliva. We adhered to the latest International Conference of Harmonization (ICH) guideline M10 on bioanalytical method validation and study sample analysis, released in 2023 [24]. The developed method is selective, specific, and linear within the 2–500 ng/mL range for both MPA and MPAG, with no carry-over and matrix effects. It met the acceptance criteria for within-run and between-run accuracy and precision. We proved the stability of MPA and MPAG in saliva samples in different conditions (2 h at room temperature, 18 h at 4 °C, and at least 5 months at − 80 °C) as well as after three freeze–thaw cycles, in a dry extract (16 h at 4 °C), and in the autosampler (8 h at 15 °C). We found that the analytes showed minimal adsorption onto Salivette^®^ cotton swabs and the samples may be diluted twofold if the concentrations in patients’ samples are above 500 ng/mL. We applied the method to determine MPA and MPAG in saliva samples from ten children with nephrotic syndrome, and found MPA and MPAG to be within the ranges of 4.6–531.8 ng/mL and 10.7–183.7 ng/mL, respectively.

Our LC–MS/MS method was linear within the 2–500 ng/mL range for both MPA and MPAG, with an LLOQ of 2 ng/mL. Wiesen et al. [20] reported linearity in a slightly narrower range (5–400 ng/mL) and different LLOQ for MPA (1.6 ng/mL) and MPAG (5 ng/mL). The lowest LLOQ reported was 1 ng/mL by Brooks et al. [15], while Shen et al. [19] and Mendonza et al. [18] reported LLOQs similar and slightly higher than ours. Ferreira et al. [17] set higher LLOQs for both MPA and MPAG (5 ng/mL and 10 ng/mL, respectively), while the ULOQ values for MPA and MPAG in their study were the same as in our method (500 ng/mL). The highest LLOQ for MPA was found by Alsmadi et al. [14] and it amounted to 150 ng/mL. As the highest saliva MPA concentration in samples collected from children included in our study was 532 ng/mL, it seems unnecessary to apply the wider concentration range, up to 2000 ng/mL, as we did in our previous study [23].

We used MPA-d3 as the IS, but only for MPA. Labeled isotopes such as IS are recommended for MS analysis [24], as they are not present in biological samples. However, in our study, MPAG-d3 could not be used for MPAG due to interference at the MPA retention time and transition. MPAG-d3 as IS should be used constantly at high concentrations, regardless of the sample type (calibration curve or biological samples with variable MPA content), therefore, it would interfere with MPA and increase its response. Moreover, the calibration curve was adopted to the 2–500 ng/mL range to avoid the interference of MPAG at MPA retention time. To determine MPAG the most precisely, we constructed the calibration curve for MPAG by plotting the MPAG peak area against its nominal concentration. We did not prepare the calibration curve samples separately for MPA and MPAG as in biological samples MPA and MPAG are both present simultaneously. In the literature, Ferreira et al. [17] did not use a labeled isotope as IS, opting for ketoprofen instead. Wiesen et al. [20], who also determined MPAG along with MPA, used isotopically labeled [^13^C, ^2^H_3_]-MPA as IS for both analytes and detected an additional signal indicating a breakdown of MPAG to MPA under electrospray ionization conditions. This transition (321.14 > 207.11) is equal to the second transition for MPA in our study (321.2 > 206.9). The authors found that the isotope co-eluted with MPA, and the MPAG signal was separated from the MPA peak which allowed to avoid the chromatographic interference between both analytes [20]. Similarly, Mendonza et al. [18], who did not analyze MPAG in saliva, observed some degree of insource fragmentation of MPAG to MPA. The result was similar to our study, i.e., the chromatogram showed traces of MPA at the retention time of MPAG. In our study, we noted a negligible peak (< 20% of MPA response at LLOQ) from MPAG in the MPA transition chromatogram but at the MPAG retention time.

Our LC–MS/MS method is selective and specific with no observed carry-over or matrix effects. Matrix effect could suppress or enhance the MS signal and affect the precision and accuracy negatively [29], therefore it is important to use several blank matrices to validate the method. Our method is also suitable for patients taking concomitant medications such as paracetamol, voriconazole, itraconazole, ketoconazole, and amlodipine as these drugs did not interfere with the determination of MPA, MPAG, and IS. Antifungal agents are commonly used after solid organ or hematopoietic stem cell transplantation, therefore, our method may be applied also in patients treated with MMF as an acute rejection prophylaxis.

Regarding sample stability, literature data suggest storing saliva samples at 4 °C for 3–6 h before further preparation for analysis [30]. We have demonstrated that saliva samples could be stored at 4 °C for 18 h, 2 h at room temperature, and at least 5 months at − 80 °C without the change of the analytes concentrations. For comparison, Ferreira et al. [17] showed stability of saliva samples at room temperature for 8 h and at − 80 °C for 3 months, while Brooks et al. [15] found stability of MPA in both, saliva and plasma matrices, for at least 2 years when stored at − 20 °C. Wiesen et al. [20] observed no significant decreases in MPA and MPAG concentrations in saliva and serum samples after storage at room temperature for 24 h. In our study, the Salivette^®^ probes were centrifuged immediately after collection, therefore, we did not extend the time for checking the stability at room temperature. In opposition to Ferreira et al. [17], we have proved the stability of MPA and MPAG in saliva samples after three cycles of freezing and thawing. We observed longer stability of the analytical samples stored in an autosampler (16 h vs. 12 h), however, the samples in our study were kept at 15 °C, whereas in the Ferreira et al. study the temperature was not defined. Similar results to ours were obtained by Shen et al. [19], who proved up to 15 h autosampler stability of the analytical samples if kept at 4 °C. Additionally, we have demonstrated the 16 h stability of MPA and MPAG in dried residue when stored at 4 °C, enabling the preparation of samples on one day and analysis on the next day.

For saliva collection, we used Salivette^®^ devices with cotton swabs, which in our opinion, are more comfortable for children. Other authors used passive drool [18, 20] or Salivette^®^ devices [15, 17, 20, 22], however, some of them specified that they used synthetic swabs [15, 22]. There is debate about whether saliva obtained with Salivette^®^ is stimulated or unstimulated. We believe that it is challenging for children not to chew the swab while keeping it in the mouth, hence we assumed that the saliva obtained in our study was stimulated. Generally, stimulation has some advantages. First of all, it increases saliva secretion, allowing the collection of a larger sample volume [31]. Secondly, it reduces viscosity, making it easier for pipetting [32]. Thirdly, the stimulation method also offers the advantage of greater patient comfort and a faster sample collection procedure, enabling the collection of biological material from a larger group of individuals [33]. Mechanical stimulation is the most effective method of saliva collection. Additionally, unlike chemical stimulation, it does not cause pain, which is why it is commonly used in children [31, 34]. Its use is, however, limited to lipophilic substances, such as fentanyl, which may undergo adsorption onto the swab. Before proceeding with the analysis, the extent of analyte adsorption should be estimated by examining its recovery from the swab. It is considered acceptable for adsorption to be below 15%, and this value is adopted from studies of precision and accuracy [34]. Our results indicated that MPA and MPAG were not adsorbed onto the Salivette^®^ cotton swabs. For recovery analysis, we incubated the Salivette^®^ with cotton swabs spiked with MPA and MPAG for 5 min at 37 °C to simulate the conditions of saliva collection (1 min in the mouth). In the hospital, the Salivette^®^ tubes were immediately centrifuged after collection, therefore we did not incubate the samples longer. In future studies, if the Salivette^®^ tubes are not centrifuged immediately, the level of MPA and MPAG adsorption onto the cotton swabs should be established for a time span longer than 5 min. Another limitation of the stimulated sampling is that it may affect saliva composition and, consequently, drug concentration. Therefore, the selection of an appropriate saliva collection method is important [15]. Wiesen et al. [20] observed excellent recovery of MPA and MPAG from synthetic swabs (> 90%), however, they incubated the samples for 3 h and 6 h at room temperature before analysis. Some authors claimed that synthetic swabs are better than cotton due to significant interference or low recovery of several substances, such as 17-hydroxyprogesterone, testosterone, and insulin [35].

In our study, using the developed method, we have determined saliva MPA and MPAG concentrations within 12 h after MMF administration in ten children with nephrotic syndrome. So far, all of the literature data included adults and children after renal transplantation [15, 17–20, 22] mostly with sparse sampling: 0, 1 h [14]; 0, 0.5, 2 h [20]; 0, 1, 2, and 4 h [22]; 0, 0.5, 1, 1.5, 2, and 12 h [17] post-dose. Only one study included pediatric patients [14], while another included only one child [20]. The range of MPA saliva concentrations in our study was within 4.6–531.8 ng/mL, whereas other authors reported the ranges of 1–819 ng/mL [15], and 2.6–220.4 ng/mL [18]. Although we observed MPA concentration above 500 ng/mL only in one child, we performed the dilution integrity test and confirmed that if MPA and MPAG concentrations exceed the ULOQ, the saliva samples may be diluted twice to fit the calibration curve. MPA concentrations varied in our study (%CV range, 62–208%), similar to Brooks et al. study [15] in renal transplant recipients treated with enteric-coated mycophenolate sodium. Our study found a roughly threefold higher MPA C_max_ (145.6 ng/mL) than Brooks et al. [15] (45 µg/L). The mean MPA C_2_ was 53.8 ng/mL in our study, similarly to renal pediatric patients in other studies (57 ng/mL) [20]. In children with nephrotic syndrome, we observed similar median MPA AUC_0–12_ to those observed by Ferreira et al. [17] in kidney transplant patients treated with MMF (339 ng h/mL vs. 333.1 ng h/mL). On the other hand, Brooks et al. [15] reported a lower median MPA AUC_0–12_ of 216.2 ng h/mL (the range 126.6–592.6 ng h/mL). Although the children were not receiving cyclosporine, we observed MPA C_max2_ in saliva within 4–12 h after MMF administration in all but one child. A similar observation was reported by Shen et al. [19], who noted MPA C_max2_ in saliva from healthy volunteers and kidney transplant patients within 6–18 h after MMF administration.

The results obtained with LC–MS/MS and HPLC-FLD methods appear to be comparable, with lower MPA concentrations determined with the HPLC-FLD method. However, it is important to emphasize that the sample size was too small to fully assess the correlation between the two methods.

The novelty of our study lies in the determination of saliva MPAG, as there is scarce literature data on this topic, especially in children with nephrotic syndrome. We found saliva MPAG concentration and AUC_0–12_ values to be within the ranges of 10.71–174.76 ng/mL and 228.12–1251.91 ng h/mL, respectively. In Wiesen et al. study [20], MPAG C_2_ determined in one child was half (47.39 ng/mL) compared to the mean C_2_ in our study (86 ng/mL), whereas the median saliva MPAG AUC_0–12_ in adult renal transplant recipients in Ferreira et al. study [17] was higher than in our study (784.2 ng h/mL vs. 535.3 ng h/mL). Based on the %CV, we observed more variable concentrations of saliva MPA than saliva MPAG.

The limitation of our study is that the children were not restricted from flossing or brushing their teeth, eating, or drinking 30 min before saliva collection due to the multiple samples collection within 12 h. The second limitation is that blank saliva samples were collected from healthy adults, not children.

As MPA and MPAG monitoring in saliva seems simple and accessible, further studies should define the target values for saliva MPA and MPAG AUC_0–12_ in children with nephrotic syndrome. At the same time, the correlation between saliva concentrations of MPA and MPAG and their plasma levels should be investigated in this group of patients, as some studies confirmed MPA plasma–saliva correlation [14, 18–20], while others have reported contradictory results [15, 22].

## Conclusion

To conclude, the evaluated LC–MS/MS method met the validation requirements for MPA and MPAG determination within the 2–500 ng/mL range. The method is selective and specific, exhibiting no carry-over and matrix effects. Saliva samples containing MPA and MPAG may be stored for 2 h at room temperature, for 18 h at 4 °C, and at least 5 months at − 80 °C. MPA and MPAG were stable in saliva after three freeze–thaw cycles, in dry extract for 16 h at 4 °C and for 8 h in autosampler at 15 °C. MPA and MPAG were not considerably adsorbed onto Salivette^®^ cotton swabs, and samples with drug concentrations above 500 ng/mL may be diluted twice. In children, MPA and MPAG concentrations were within 4.6–531.8 ng/mL and 10.7–183.7 ng/mL, respectively. The evaluated method is suitable for use in children with nephrotic syndrome, however, further studies focusing on the correlation between saliva MPA and total and free plasma MPA and MPAG, and its potential contribution to MPA TDM, are required.

### Supplementary Information

Below is the link to the electronic supplementary material.

Supplementary file1 (PDF 70 KB)

Supplementary file2 (PDF 40 KB)

Supplementary file3 (DOCX 15 KB)

Supplementary file4 (XLSX 13 KB)

## Data Availability

The datasets generated during and/or analyzed during the current study are available from the corresponding author upon reasonable request.

## Abbreviations

AUC_0–12_: Area under the concentration–time from 0 to 12 h curve
CI: Confidence interval
C_max_: Maximum concentration
C_max2_: Secondary maximum concentration
C_trough_: Concentration before the administration of the next dose
C_1_–C_12_: Concentrations determined at definite times from 1 to 12 h after dosing
%CV: Coefficient of variation
ESI+: Positive electrospray ionization mode
ESI−: Negative electrospray ionization mode
FLD: Fluorescence detection
HPLC: High-performance liquid chromatography
ICH: International Conference of Harmonization
IS: Internal standard
LC–MS/MS: Liquid chromatography-tandem mass spectrometry
LLOQ: Lower limit of quantification
MMF: Mycophenolate mofetil
MPA: Mycophenolic acid
MPA-d3: Deuterated mycophenolic acid
MPAG-d3: Deuterated mycophenolic acid glucuronide
MPAG: Mycophenolic acid glucuronide
QC: Quality control
SD: Standard deviation
SECS: Salivary Excretion Classification System
TDM: Therapeutic drug monitoring
T_max_: Time to reach maximum concentration
T_max2_: Time to reach secondary maximum concentration
ULOQ: Upper limit of quantification
UPLC: Ultra-performance liquid chromatography
