# Correction: Liquid chromatography–tandem mass spectrometry method for mycophenolic acid and its glucuronide determination in saliva samples from children with nephrotic syndrome

**DOI:** 10.1007/s43440-024-00617-1

**Published:** 2024-06-25

**Authors:** Joanna Sobiak, Matylda Resztak, Weronika Sikora, Jacek Zachwieja, Danuta Ostalska-Nowicka

**Affiliations:** 1 Department of Physical Pharmacy and Pharmacokinetics, Poznan University of Medical Sciences, 3 Rokietnicka Street, 60-806 Poznan, Poland; 2 Department of Pediatric Nephrology and Hypertension, Poznan University of Medical Sciences, Poznan, Poland

**Correction: Pharmacological Reports (2024) 76:600-611**

10.1007/s43440-024-00574-9

In this article ref. 25 was duplicated. Due to this, further reference numbers are all assigned incorrectly. That is, in Results section, at the fifth line in the paragraph start with “Matrix effects, often caused by alterations”, “[25, 25]” should have been “[25, 26]”.

Further reference numbers, [27] to [34] should have been [28] to [35].

In addition to this, in Discussion section, at the twelve line in the paragraph start with “In our study, using the developed method”, “2.6-220.4 ng/mL [35],” should have been deleted.

The original article has been corrected.

