# Supplementary figures and images for: Liquid chromatography–tandem mass spectrometry method for mycophenolic acid and its glucuronide determination in saliva samples from children with nephrotic syndrome
Source: Pharmacol Rep. 2024 Mar 15;76(3):600–11. doi: 10.1007/s43440-024-00574-9 (PMC11126467; doi:10.1007/s43440-024-00574-9)

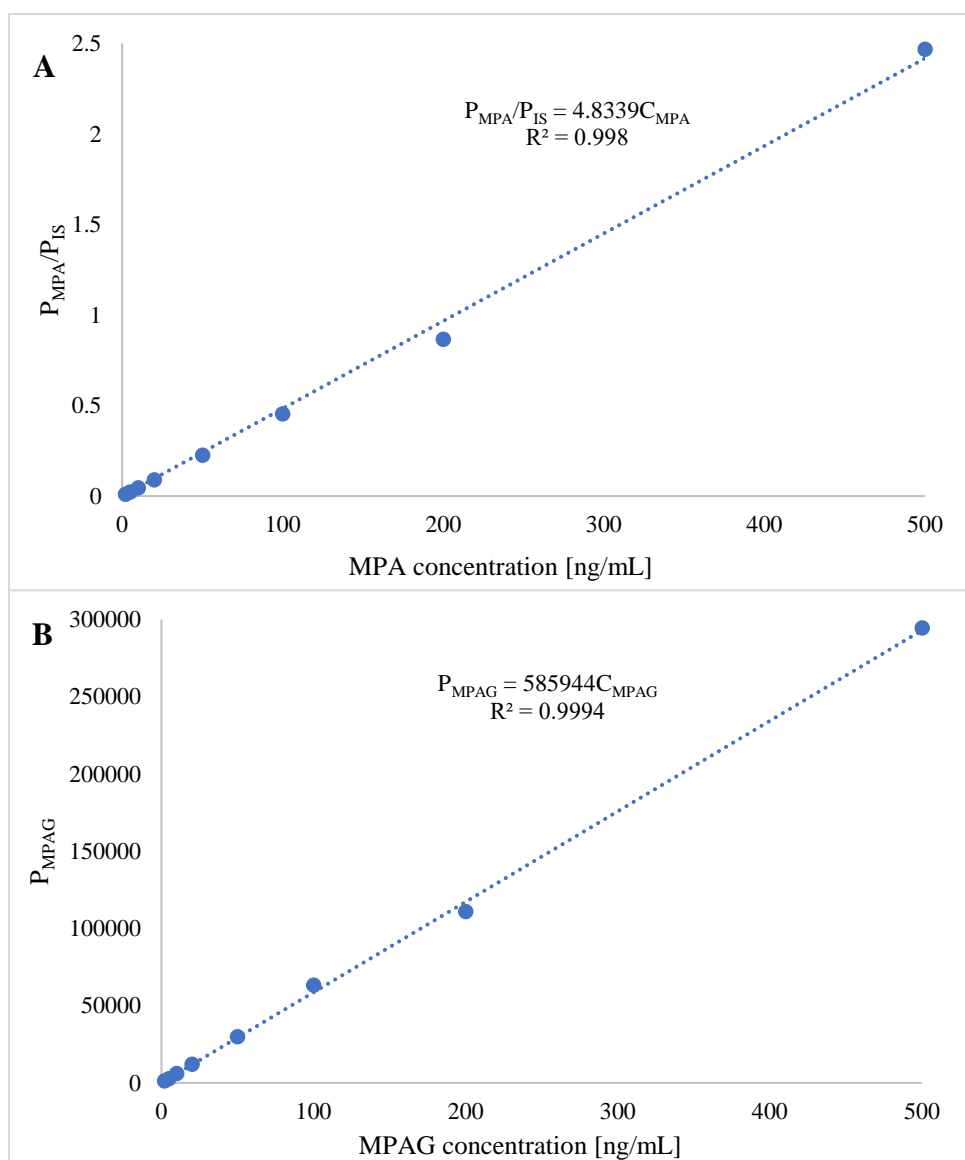

Supplement: Supplementary file 1 — Supplementary file1 (PDF 70 KB) [file 43440_2024_574_MOESM1_ESM.pdf]

**A****Bland-Altman analysis**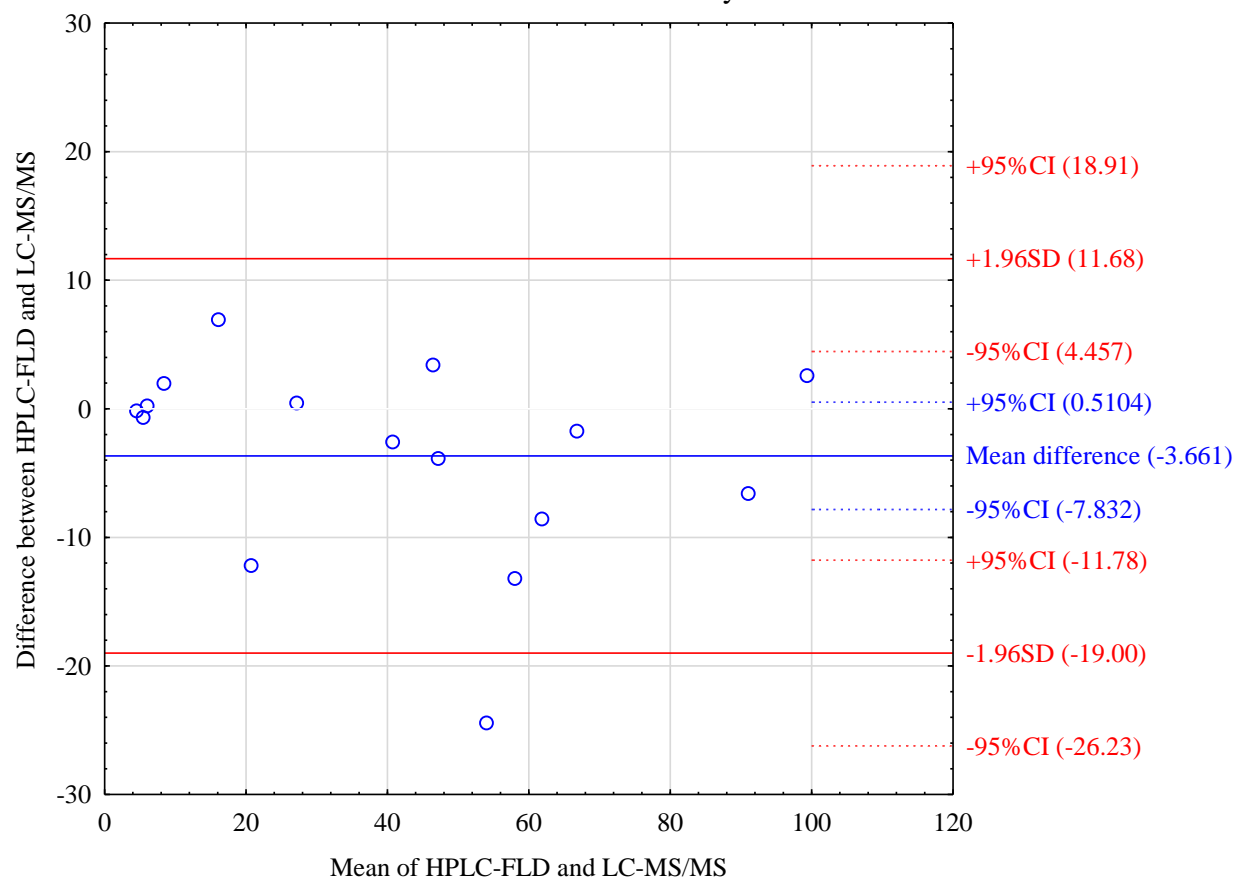**B****Passing-Bablok regression analysis**  
**HPLC-FLD = 0.1539+0.9345 LC-MS/MS**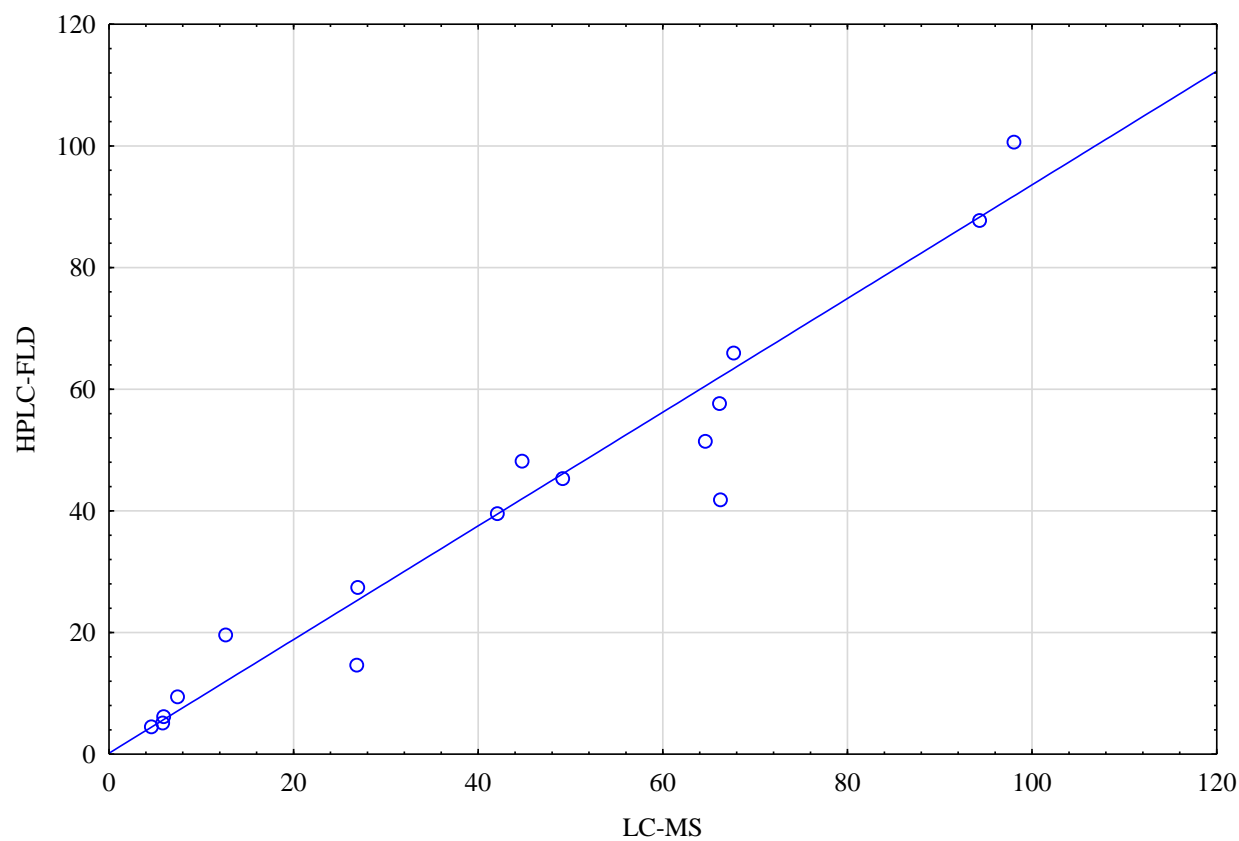

Supplement: Supplementary file 2 — Supplementary file2 (PDF 40 KB) [file 43440_2024_574_MOESM2_ESM.pdf]
