# Supplementary material for: Liquid chromatography–tandem mass spectrometry method for mycophenolic acid and its glucuronide determination in saliva samples from children with nephrotic syndrome
Source: Pharmacol Rep. 2024 Mar 15;76(3):600–11. doi: 10.1007/s43440-024-00574-9 (PMC11126467; doi:10.1007/s43440-024-00574-9)
Supplement: Supplementary file 3 — Supplementary file3 (DOCX 15 KB) [file 43440_2024_574_MOESM3_ESM.docx]

**Supplementary Figure S1.**

The calibration curves for mycophenolic acid (MPA, **A**) and mycophenolic acid glucuronide (MPAG, **B**). The MPA calibration curve was constructed using the ratio of the MPA peak area (P_MPA_) to the internal standard (IS, deuterated MPA) peak area (P_IS_), plotted against the MPA nominal concentrations. The equation of the calibration curve, presented with its square correlation coefficient, was P_MPA_/P_IS_ = 4.8339∙C_MPA_ (R² = 0.998). For MPAG, the calibration curve was constructed by plotting the MPAG peak area (P_MPAG_) against the MPAG nominal concentrations. The equation for this calibration curve, with its square correlation coefficient, was P_MPAG_ = 585944∙C_MPAG_ (R² = 0.9994).

**Supplementary Figure S2.**

Comparison of LC-MS/MS and HPLC-FLD methods for measuring mycophenolic acid (MPA) concentrations in saliva (n = 16). In the Bland-Altman plot (**A**), the mean difference, along with its 95% confidence interval (CI) was -3.661 (CI from -7.832 to 0.5104). The lower limit of agreement was -19.00 (CI from -26.23 to -11.78), and the upper limit of agreement was 11.68 (CI from 4.457 to 18.91). The empty circles represent the paired samples, the solid blue line indicates the mean difference and its 95% CI, the dashed blue lines represent the limits of agreement with their 95% CIs, and the line of equality, which is 0, is not included in the plot. In the Passing-Bablok regression analysis (**B**), the slope, along with the 95% CI, was 0.9345 (CI from 0.8047 to 1.0818), and the intercept, with the 95% CI, was 0.1539 (CI from -5.7301 to 4.997). The empty circles denote the paired samples, and the solid blue line represents the regression line.
